# Validation of a modified visual analogue scale to measure user-perceived comfort of a lower-limb exoskeleton

**DOI:** 10.1038/s41598-023-47430-z

**Published:** 2023-11-22

**Authors:** Mohammed Mohammed El Husaini, Axl Maberry, Anne E. Martin

**Affiliations:** Department of Mechanical Engineering, Pennsylvania State University, University Park, PA, 16802 USA

**Keywords:** Biomedical engineering, Mechanical engineering

## Abstract

User perceived exoskeleton comfort is likely important for device acceptance, but there is currently no validated instrument to measure it. The Visual Analogue Scale (VAS) is an existing tool to measure subjective human feedback by asking the user to mark a point on a line with each end of the line representing an opposing anchor statement. It can be modified to show the previous response, allowing the subject to directly indicate if the current condition is better or worse than the previous one. The goal of this study was to determine how well the modified VAS could measure user-perceived comfort as the exoskeleton control parameters were varied. To validate the survey, 14 healthy subjects walked in a pair of ankle exoskeletons with approximately ten distinct sets of control parameters tested in a prescribed order. Each set of control parameters was tested twice. After each trial, user-perceived comfort was measured using a two-question VAS survey. The repeatability coefficient was approximately 40 mm, similar to the total range of responses. The results were also inconsistent, with relative rankings between consecutive pairs of conditions matching for approximately 50% of comparisons. Thus, as tested, the VAS was not repeatable or consistent. It is possible that subject adaptation within the trial and over the course of the experiment may have impacted the results. Additional work is needed to develop a repeatable method to measure comfort and to determine how perceived comfort varies as subjects’ gain exoskeleton experience.

## Introduction

Exoskeletons have the potential to enhance rehabilitation in healthcare settings and to reduce injury in industrial settings. As the population ages, gait-related problems are expected to increase^1^. These problems tend to reduce quality of life^1^ and increase the risk of secondary conditions^2^, motivating the development of novel rehabilitation methods such as exoskeletons^3,4^. In physically-demanding industries, exoskeletons can be used to reduce muscular effort^5^. This in turn may reduce the likelihood of injury and the associated costs^6^. Given the high rates (> 20%) of injury in the construction industry^7^, nursing^8^, and other fields with highly-loaded repetitive movements^9^, there is a great need for such assistive devices.

For exoskeletons to be adopted, they need to be effective as well as meet many subjective criteria such as being comfortable and not generating negative social perceptions^10–14^. However, subjective criteria are only just beginning to be studied, particularly for lower-limb exoskeletons. While there is more work on comfort and user perception for upper-limb and back exoskeletons, there is not a standard, validated method to measure (dis)comfort^15^. Much of the work measuring comfort has focused on the physical design of the device^11,16^ and ignored how the exoskeleton controller impacts comfort. A few groups have optimized for user preference in lower-limb exoskeleton controllers^17–21^ and prosthetic stiffness^22–27^, but these studies did not directly consider comfort. While comfort is likely an aspect of preference, it is not necessarily equivalent to it, although comfort and preference are often assumed to be synonymous in the literature e.g.,^18,28,29^. In contrast to the previous studies, our study directly asked how comfortable the exoskeleton felt.

Unfortunately, measuring subjective feelings is difficult. Although the person may know exactly how they feel, communicating that information can be difficult due to the difficulty in verbalizing subjective evaluations of comfort^24,26,30^. Thus, methods such as Visual Analogue Scale (VAS) surveys^15,20,30–33^ are used to measure subjective feelings. A standard VAS utilizes two opposing anchor statements that describe the measured variable^34^. They are typically presented as a horizontal line with a length of 100 mm^35^, and the subject responds by marking a point on the line. The response is then analyzed as a continuous numerical value. The VAS can be modified to include the previous response, thereby allowing the subject to make a direct comparison^34,36,37^. This technique allows the data to be analyzed as pairwise comparisons between consecutive conditions. However, very few studies have used VAS surveys for lower-limb exoskeletons^20^.

Instead, some studies have used variations on forced choice comparisons to find the most preferred exoskeleton controller or prosthesis setting^17,18,21,23,24,26,27^. These methods produce pairwise rankings but do not produce an absolute measure of the feeling. Other studies have had subjects self-tune device parameters to specify their most preferred condition^19,22,25,28^. This method does not produce a measure of the relevant subjective feeling; it only finds the associated optimal parameters. In theory, a modified VAS should measure both the relative comparison between consecutive conditions and provide an absolute value of comfort^34^, providing the most flexibility for later data analysis. Standard VAS surveys can reliably measure footwear comfort^31^ and pain^32,38–42^ but have not yet been validated for lower-limb exoskeleton comfort.

A valid measurement tool needs to produce a similar response when the same condition is tested on the same person multiple times^43^. In other words, it needs to be repeatable. This is typically quantified using the repeatability coefficient (*RC*), with lower values indicating better repeatability^43,44^. The *RC* for pain using a standard 100 mm VAS scale is generally 12–20 mm^42,45,46^. The primary objective of this study was to measure the *RC* of a VAS for exoskeleton comfort.

Traditional validation methods for the VAS implicitly assume that the subject’s internal perception of the phenomena does not change as the experiment progresses. This may not be a valid assumption when measuring exoskeleton comfort, which would then make the test–retest approach unsuitable. Because most subjects will have very limited prior experience with exoskeletons, they may not have a good sense of what the maximum anchor feels like at the start of the experiment. As they gain more experience, the interpretation of the maximum anchor could change, causing responses to drift and lowering the repeatability of the survey^30,36^. Further, as the experiment progresses, subjects may become better at walking with the exoskeleton^47,48^, changing which controller feels most comfortable. If subjects become fatigued, perceived comfort could systematically decrease as the experiment progresses. There is some evidence that adaptation occurs when measuring footwear comfort because shoes worn prior to a comfort measurement affect the results^31^, implying that perceived comfort changed over time. Thus, the data needs to be checked for potential user adaptation to help interpret the validation results.

Therefore, the primary objective of this paper was to quantify the repeatability of a modified VAS survey that measured the user-perceived comfort of an ankle exoskeleton controller. To further investigate the consistency of the survey, the VAS responses were converted into relative rankings between consecutive pairs of experimental trials, and the consistency of the rankings were quantified. Because these analysis techniques assume that the user’s internal perception of comfort does not change over the course of the experiment, preliminary qualitative results on potential user adaptation are given.

## Results

In this study, young healthy adults walked in a pair of ankle exoskeletons for approximately 20 3-min trials. After each trial, subjects answered a two-question 100 mm VAS survey that showed the response for the previous trial. The first question (Q1) was: “How comfortable are you?” and the second question (Q2) was: “How helpful is the exoskeleton at toe-off?”. Each subject experienced approximately 10 unique exoskeleton control parameters in a prescribed order—all 10 parameter sets were tested once in the first cycle and then all 10 parameter sets were tested a second time in the second cycle. Prior to the experiment, subjects had not used an exoskeleton. Our analysis aimed to determine if user responses were repeatable between the two cycles.

### Initial analysis

The two VAS questions were strongly correlated with a Pearson correlation coefficient of 0.79. Correlations for most subjects were similar, with correlations ranging from 0.56 to 0.92, except for a single subject with a correlation of $-0.10$ (Supplemental Fig. 1).

The median range of VAS responses was 40 mm for Q1 and 42 mm for Q2, although there was significant between-subject variability (ranges of 13–87 mm for Q1 and 23–100 mm for Q2, Supplemental Table 1). Thus, most subjects did not utilize the entire range of the VAS.

**Figure 1:**
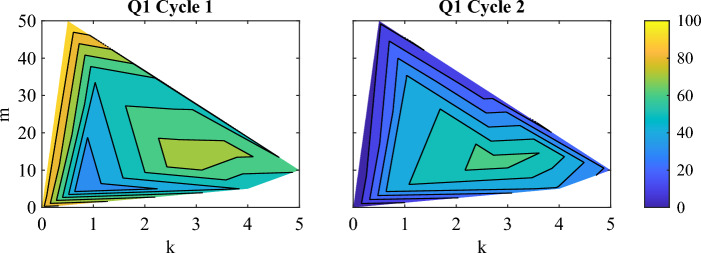
Comparison of normalized Q1 answers (Eq. 4) between cycles for one representative subject. The exoskeleton control parameters are *k* (effective stiffness) and *m* (rate of reference position change). For this subject, they found low *k* most comfortable in cycle 1. In cycle 2, they found moderate *k* and moderate *m* most comfortable. In 6 out of 14 subjects, the area of maximum comfort shifted towards more moderate *k* and *m* in the second cycle compared to the first cycle.

Subject answers were not consistent between cycles. When each cycle’s data was plotted separately, 11 out of the 14 subjects had substantially different comfort contours between cycle 1 and 2 (Fig. 1, Supplemental Fig. 2). For 6 subjects, their most comfortable parameters shifted to more moderate parameter values in cycle 2. The remaining 5 subjects with changes all altered their preferences differently. When the VAS score was plotted versus trial number, there were no consistent trends such as a consistent decrease in comfort as the experiment progressed (Supplemental Fig. 3).

### Repeatability

**Figure 2:**
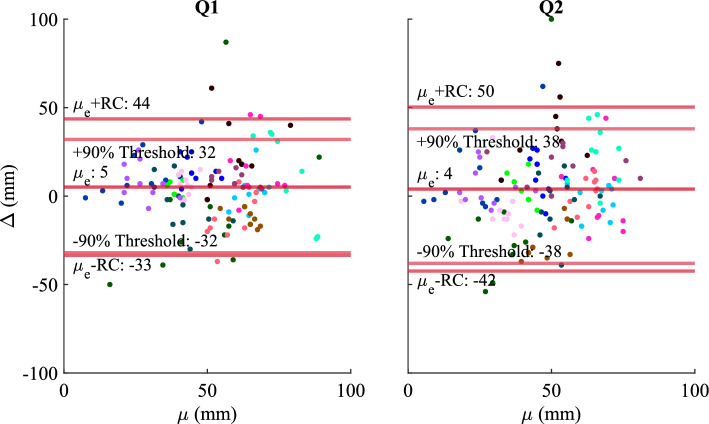
Difference in VAS scores between cycles ($\Delta$) versus mean VAS response ($\mu$) per condition and subject. Each dot color represents a different subject. There were significant deviations among responses signifying poor repeatability. The repeatability coefficient of Q1 was slightly smaller than Q2, indicating that Q1 had better repeatability.

**Table 1:** Results from the repeatability analysis when all subjects were analyzed together.

	*RC* (mm)	${\mu }_\Delta$ (mm)	${\sigma }_\Delta$ (mm)	$\Delta {<10 \text { mm}}$ (%)	$\Delta {<20 \text { mm}}$ (%)	90% threshold (mm)
Q1	39	5	19	47	75	32
Q2	46	4	23	40	65	38

The table contains the repeatability coefficient (*RC*), signed mean difference between cycles ($\mu _\Delta$), standard deviation of the difference between cycles ($\sigma _\Delta$), percent of trials with between cycle differences ($\Delta$) less than 10 mm and 20 mm, and the 90% threshold. The 90% threshold was defined as the difference magnitude for which 90% of responses had absolute differences less than the threshold. Although the (*RC*) for both questions was large, 75% of the differences between cycles ($\Delta$) for Q1 were below 20 mm, implying that the VAS survey could measure a signal but not as well as for pain.

When all subjects were analyzed together, the *RC* was 39 mm for Q1 and 46 mm for Q2 (Fig. 2, Table 1). When each subject was analyzed individually, the median (minimum–maximum) *RC* was 33 (13–76) mm and 36 (17–85) mm for Q1 and Q2 respectively. Most subjects’ individual *RC* encompassed the majority and, in some cases, the entirety of their range of responses for each question (Supplemental Table 1).

The 90% threshold of approximately 35 mm was similar to the *RC* as expected (Table 1). The percentages of trials with between cycle differences less than 10 mm and 20 mm were 47% and 75% for Q1 respectively. Consistent with the *RC*, these percentages were larger than the corresponding percentages of 40% and 65% for Q2. Both questions had a small positive bias $\mu _\Delta$, indicating that subjects generally rated sets of parameters slightly higher during the first cycle than in the second cycle. Due to the large *RC* for both questions, the bias was insignificant.

### Relative comfort consistency

**Table 2:** The number of trials for each relative ranking arranged by cycle.

Question 1				Question 2			
Cycle 2	Cycle 1			Cycle 2	Cycle 1		
	Higher	No change	Lower		Higher	No change	Lower
Higher	20	13	12	Higher	26	9	14
No change	12	21	4	No change	10	15	11
Lower	14	10	19	Lower	15	8	17

The relative rankings were not consistent between trials, as indicated by the large number of trials in the off-diagonal table cells.

The relative comfort consistency between consecutive trials was 48% for Q1 and 46% for Q2 (Table 2). When the relative ranking changed between cycles, it had an approximately equal chance of moving to either of the two other options.

## Discussion

This work evaluated how consistently a modified VAS survey could measure user-perceived comfort of an ankle exoskeleton controller. The VAS instrument allowed explicit comparisons by showing the previous response. While multiple studies have shown that a VAS can measure footwear comfort^31^ and general pain^32,38–42^, it did not produce repeatable measurements for exoskeleton comfort. The *RC* of $\approx$ 40 mm was more than twice as large as the *RC* for pain^42,45,46^. Further, the response range had a similar magnitude as the *RC*. Together, this indicates that individual subjects did not give similar absolute scores for identical control parameters. In addition, when comparing relative comfort scores between consecutive trials, the relative rankings were not consistent between cycles. This indicates that individual subjects did not consistently find one set of exoskeleton control parameters more comfortable than another set.

The lack of consistency is somewhat surprising since previous research has indicated that people can reliably choose which exoskeleton or prosthesis condition they prefer^17–19,22–24,26,28^. While comfort and preference are often thought of as synonymous, comfort is likely only one factor that influences preference. Thus, one possibility is that preference is primarily driven by factors other than comfort, such as metabolic cost or stability, so that preference and comfort are not strongly correlated. Future work is needed to better understand what factors influence comfort and preference. Another possibility is that adaptation plays a large role in perceived comfort. Multiple studies have shown that subjects adapt to walking with an exoskeleton, including preferring more assistance as the experiment progresses^19,47^. In this study, many subjects’ most comfortable control parameters shifted to more moderate values as they gained experience. Thus, subject adaptation could explain both the inconsistent relative responses and the low repeatability. This adaptation can occur during an individual trial, as well as across the entire experiment.

In this work, within trial adaptation was supported by statements from some subjects. They stated that at first, they might have felt a difference between changes to the set of control parameters, but then they could no longer tell by the end of the trial. Thus, it appears that requiring subjects to walk for several minutes in each condition may reduce the differences in perceived comfort as subjects learn to walk well with different controller parameters. In the preference studies, subjects only took a few steps before giving their response^19,27^, which may have improved the results. One way to resolve the issue of within-trial adaptation is to reduce the duration of the trial. However, this would make measuring other biological signals more difficult, particularly steady-state metabolic cost. Another approach could be introducing the survey during the trial, which would require the subject to answer the survey as they walk. This could result in subjects stumbling or losing balance as they focus on the survey. These approaches would also only give the initial impression of comfort, not how comfortable the control condition is after extended use. Additional studies investigating how within trial adaptation influence comfort are needed. Alternatively, larger changes in control parameters between trials may improve the repeatability by creating more of a difference between conditions. It is possible that subjects could not feel a difference between some conditions^49,50^, although if this occurred, subjects should have given the same VAS score for both trials. If the survey repeatability is only acceptable for large changes in control parameters, it would indicate that large changes in perceived comfort could be measured, but not small differences.

Adaptation over the duration of the experiment also may have occurred. As discussed in the introduction, this can change the perceived maximum (or minimum) and cause drift over time. If this occurred, the responses would not be repeatable, but the relative consistency between consecutive trials should have been high. Since this was not observed, it is unlikely that this occurred. Subjects could also adapt to the physical design of the exoskeleton or become fatigued. If either of these occurred and were the primary factor driving comfort, we would expect the mean comfort for the first cycle to be substantially different than for the second cycle. We would also expect to see a general trend of increasing or decreasing comfort scores as a function of trial number regardless of how the control parameters changed between trials. However, neither of these phenomena were observed, so it does not appear perceived comfort systematically increased or decreased over the course of the experiment.

Finally, the experimental protocol could influence adaptation and the perception of comfort. Much of the previous work that validated preference had subjects self-select their most preferred condition after freely exploring the parameter space^19,22,28^. It is possible that the initially diverse exploration prior to fine-tuning quickly got subjects used to the device and the range of possibilities, making it easier for them to identify their most preferred condition. If this is true, then performing a third cycle in this experiment and calculating the repeatability between the second and third cycles could give more consistent responses. Alternatively, introducing familiarization trials with several diverse control parameters prior to data collection may improve repeatability in less time than performing a full third cycle. It is also possible that introducing a control condition before each new set of parameters^31^ could lead to more consistent answers between cycles. In theory, it would improve the subjects’ ability to recall and make comparisons while answering the survey since they would always compare their experience to the same condition. However, this would halve the number of parameters that could be tested in a given time, making it difficult to explore the exoskeleton control space fully.

The validation methods used in this paper assumed that an individual’s perceived comfort could be fully defined by the control parameters. However, if the perception of comfort changed during the course of the experiment, then that assumption was violated, and the statistical tools used were inappropriate. If adaption did occur, then comfort needs to be analyzed differently, such as with a dynamic model that includes a time component. Both the large *RC* and the inconsistent relative responses partially support a dynamic interpretation of comfort, although the standard statistical methods used here cannot be used to fully asses if this is the case. Thus, the possible temporal nature of comfort should be investigated further using methods from dynamic systems.

Overall, the VAS yielded poor validation results for measuring comfort. The repeatability analysis yielded a *RC* of 39 mm for the combined data of Q1, which was significantly larger than the *RC* of 12–20 mm for surveys designed to measure pain^42,45,46^. In addition, under 50% of the relative responses between trials were the same between cycles. The results imply that the VAS was unable to measure comfort reliably. However, this could be due to subject adaptation both within a trial and over the course of the experiment. Much more additional work is needed to determine if and how perceived comfort varies as subjects’ gain exoskeleton experience.

## Methods

### Experimental design

14 healthy subjects (12 males, 2 females, mean ± SD, age 22.6 ± 3.0 years, mass 70.1 ± 12.1 kg, and height 172 ± 11.3 cm) consented to participate in the study. The subjects had not used an exoskeleton prior to the experiment and had no neuromuscular or neurological pathologies, injuries, or illnesses affecting gait. Individuals with significant femoral retroversion (duck feet posture) were excluded from the study since it caused frequent exoskeleton collisions. The experiment was approved by the Pennsylvania State University Institutional Review Board (STUDY00012828) and conducted in accordance to relevant guidelines and regulations. All subjects provided informed consent prior to starting the experiment.

For each trial, the subject walked on a treadmill for 3-min wearing a pair of ankle exoskeletons. To account for the weight of the exoskeletons, a slow treadmill speed of $v=0.0033h$, where *h* was the subject’s height in meters, was used^51^. The exoskeleton control parameters changed every trial. After each trial, a two-question VAS survey was presented on an electronic tablet to collect user-perceived comfort responses from the subject. In addition, kinematic, EMG, and metabolic signals were recorded but not used for this analysis.

**Figure 3:**
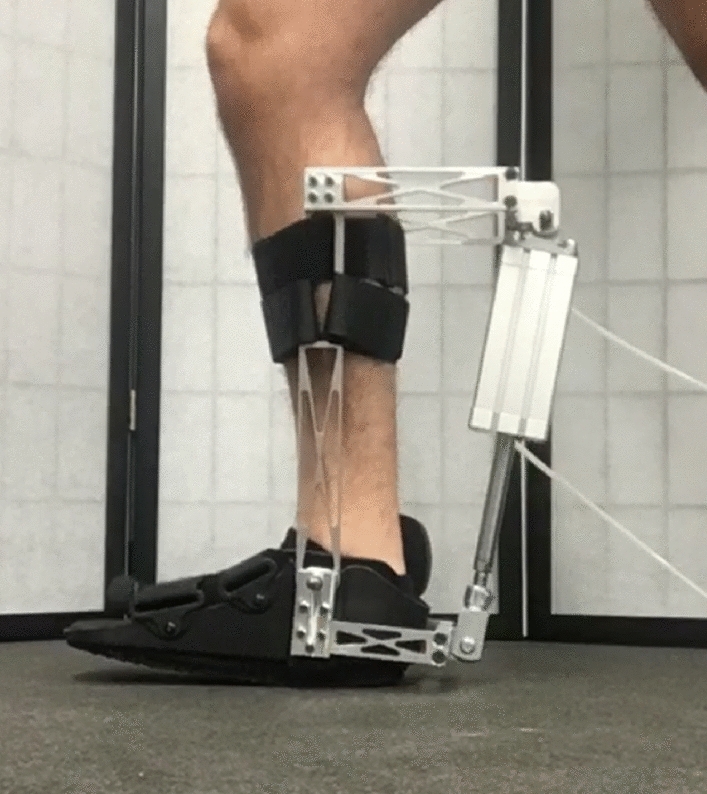
One of the two one-degree-of-freedom ankle exoskeletons. As the pneumatic cylinder actuates, it changes the ankle angle.

The 3.6 kg custom-built exoskeletons had a single pneumatically-powered degree of freedom at the ankle to allow plantarflexion and dorsiflexion^52^ (Fig. 3). To allow gait phase detection, each exoskeleton was equipped with two force sensitive resistor (FSR) sensors, one at the ball of the foot and one at the heel. Voltage thresholds for the FSRs were set manually for each subject to achieve a binary (high/low) state for each sensor to indicate if that portion of the foot was loaded. The exoskeletons were controlled using an impedance controller:

1 $$\begin{aligned} u = k_R(\theta -m_Rt), \end{aligned}$$

where $k_R$ was the virtual stiffness, $\theta$ was the current ankle angle, $m_R$ was the ramp input slope, and *t* was time. Time was reset to zero at the start of each phase. The desired ankle angle $m_Rt$ saturated at a phase-dependent reference position (Table 3). The control parameters ($k_R$, $m_R$) were normalized based on subject height and mass since it was expected that subject size would affect the magnitude of their most comfortable stiffness and ramp slope:

2 $$\begin{aligned} k&= 1000\frac{k_R}{m_sgh} \end{aligned}$$

3 $$\begin{aligned} m&= m_R\sqrt{\frac{h}{g}}, \end{aligned}$$

where $m_s$ is subject mass, *h* is subject height, and *g* is gravitational acceleration. *k* was scaled by a factor of 1000 to make the values easier to work with. The physical limits for the control parameters were found by pilot testing various parameter combinations multiple times to ensure the exoskeletons were operational for the entire trial duration. The final range for each parameter was $k \in \left[ 0,5\right] \approx \left[ 0,6 \text { {Nm}/{deg}}\right]$ and $m \in \left[ 0,50\right] \approx \left[ 0,120\text {{deg}/{s}}\right]$.

**Table 3:** Mapping between gait phase, FSR sensor readings, and desired angle saturation limit.

Gait phase	Initial stance	Middle stance	Toe-off	Swing
Front sensor	Low	High	High	Low
Back sensor	High	High	Low	Low
Saturation limit	0$^\circ$	0$^\circ$	$-20^\circ$	0$^\circ$

A negative angle indicates plantarflexion.

**Figure 4:**
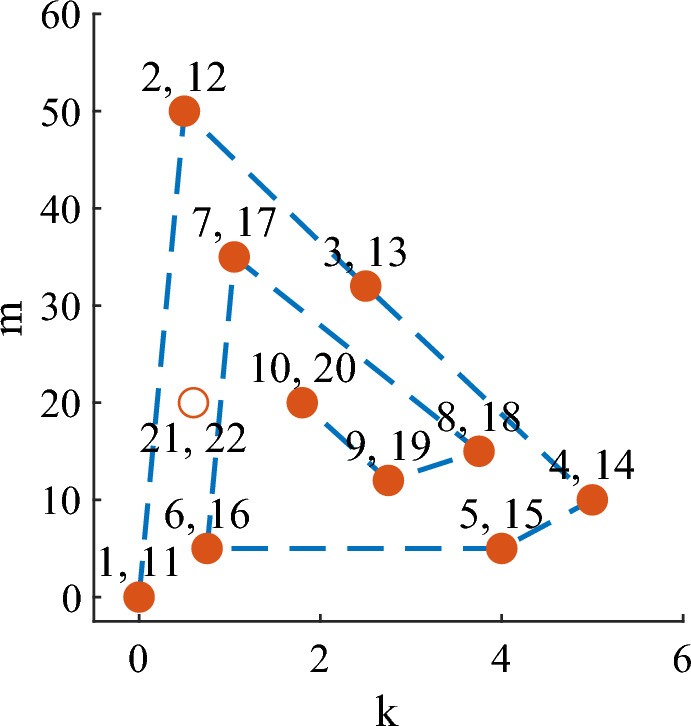
Tested control conditions. The filled circles indicate the first and second cycle parameters. The open circle is the extra set of parameters tested if there was enough time after the second cycle was completed. The numbers represent the order in which the parameters were tested.

Subjects completed at least twenty 3-min trials during the 3-h experiment. During these trials, a set of 10 control parameters were each tested twice following a roughly spiral pattern (Fig. 4). The first and second time a parameter set was tested will be referred to as the first and second cycles respectively. If there was time, subjects completed 2 additional trials for an 11th set of control parameters. Subjects were given 15-min to rest after every 7 trials to mitigate the effects of fatigue on comfort perception. The frequency and duration of the rest breaks was determined during pilot testing.

**Figure 5:**
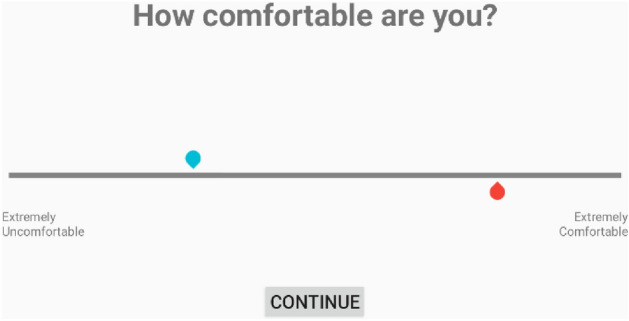
Example VAS question and response. The blue handle indicated the previous response and could not be moved. The red handle was moveable, and the subject was free to place it anywhere on the line.

After each trial, a two-question VAS survey was used to measure user-perceived comfort. For the first trial, a standard 100 mm VAS was used. Starting with the second trial, the 100 mm VAS displayed the subject’s response for the previous trial. This allowed the subject to more easily place their answer relative to their last response (Fig. 5)^34^. Pilot testing indicated that this had the potential to improve response consistency by assisting subjects in recalling their experience^34,36,53^. Prior to displaying the VAS questions, the subject was asked a preliminary question about whether he/she felt a difference between the current and previous trials. If the subject answered no, the VAS survey was skipped, and his/her previous responses were assigned for the current trial.

The VAS survey consisted of two questions: The first question (Q1) was: “How comfortable are you?” and the second question (Q2) was: “How helpful is the exoskeleton at toe-off?”. Before starting the experiment, toe-off was explained to subjects as: “One of the questions includes the term toe-off. Toe-off describes the portion of the step just before your foot leaves the ground. During this stage, you push off the ground using the ball of your foot and your toes. Hence the term, toe-off.” Q2 was meant to illicit a response more specific to changes in *m* since the exoskeleton assistance at toe-off is directly related to the magnitude of *m*. Therefore, we anticipated that Q2 would have less variability since Q2 was more specific than Q1. If Q1 yielded inconsistent responses but the questions were correlated, Q2 could be used as a proxy for Q1. Additional questions, including those focusing on a single body part, were considered during pilot testing. The two chosen questions appeared to perform the best.

When interpreting the correlation between questions, a “halo effect” (order bias^54^) could cause subsequent questions on a survey to depend on the first question posed^55^. The halo effect could lead to false conclusions about the correlation between questions since they would be answered similarly. This phenomenon was encountered during pilot testing, possibly due to mental fatigue, especially toward the end of the experiment. To mitigate this, the questions were asked in a random order^54^.

### Initial analysis

Prior to validating the survey, several preliminary measures were determined. The correlation between questions was found for all subjects together and for each subject individually. The correlation was quantified using the Pearson correlation coefficient. In addition, the range of responses was found for both questions.

Two types of plots were created to qualitatively analyze how comfort changed as the experiment progressed. In one set of plots, comfort was estimated across the range of tested control parameters using linear interpolation. Specifically, Matlab’s fit function was used with the linearinterp option. These plots were created for each subject and cycle individually and for each subject when combining the data from both cycles. To aid in visualization, the experimental data was scaled linearly using min/max feature scaling:

4 $$\begin{aligned} Q_{scaled} = \frac{Q-Q_{min}}{Q_{max}-Q_{min}}\times 100. \end{aligned}$$

where $Q_{scaled}$ was the scaled response for that set of control parameters, *Q* was the original response, and $Q_{min}$ and $Q_{max}$ were the minimum and maximum responses over all experimental trials for that subject. These plots allowed qualitative analysis of how the most comfortable control parameters changed as the experiment progressed. In the second set of plots, the VAS answers were plotted versus trial number. The plots were visually checked for consistent trends. For example, consistently decreasing VAS scores may indicate that the subject was becoming fatigued and therefore found walking in the exoskeleton increasingly difficult and uncomfortable.

### Repeatability analysis

The repeatability of each question was quantified using^44^:

5 $$\begin{aligned} RC=2.77s_w, \end{aligned}$$

where $s_w$ is the within-condition standard deviation, estimated using the root mean square error from a one-way ANOVA. In the ANOVA, the factor was the subject, the levels were the control parameter sets, and the dependent variable was the VAS answers for one question. The *RC* was determined for all subjects combined and for each subject individually. For a given set of control parameters, the cycle 2 answer was expected to be within $\pm RC$ of the cycle 1 answer in 95% of the cases.

To summarize the results of the repeatability analysis, Bland-Altman plots were constructed^44^. In these plots, the between-cycle difference $\Delta =c_1-c_2$ was plotted against the mean VAS score $\mu ={(c_1+c_2)}/{2}$, where $c_1$ is the response to a condition during the first cycle, and $c_2$ is the response to the same set of exoskeleton control parameters during the second cycle.

The percentage of trials with $|\Delta | < 10$ mm and $|\Delta | < 20$ mm were calculated to quantify the number of cases that had acceptable consistency. The bounds of 10 mm and 20 mm were chosen because the *RC* for pain is between 12 and 20 mm^42,45,46^. In addition, the 90% threshold was calculated. The 90% threshold was defined as the difference magnitude for which 90% of responses had absolute differences less than the threshold.

### Relative comfort consistency

To assess how consistently subjects compared consecutive pairs of conditions, the VAS responses were converted into relative rankings. Specifically, the VAS responses for each trial were relabeled as “higher”, “lower”, or “no change” from the previous trial. The tolerance for “no change” was set to 2 mm, meaning that responses with a difference below the tolerance were considered the same. For example, if a subject responded 60 for trial 1, 70 for trial 2, and 69 for trial 3, the relative response of trial 2 was higher than trial 1 but the same as trial 3. The 2 mm threshold was chosen because there were multiple cases in which one question had a large difference and the other question had a tiny, but non-zero, difference from the previous trial’s answer. Thus, it appeared that the subject only wanted to change their answer for one question but did not give exactly the same answer for the other question. The 2 mm threshold accommodated this.

To quantify consistency, the relative rankings between cycles 1 and 2 were compared (Table 4). For example, the relative ranking between trials 1 and 2 was compared to the relative ranking between trials 11 and 12. If the relative rankings for a given condition were the same, then that condition was consistent. The percentage of consistent conditions over all subjects was found for each question.

**Table 4:** Consistency was evaluated by comparing the relative response from cycles 1 and 2.

Cycle 2	Cycle 1		
	Higher	No change	Lower
Higher	$\checkmark$	×	×
No change	×	$\checkmark$	×
Lower	×	×	$\checkmark$

A $\checkmark$ indicates a consistent relative response, and an × represents an inconsistent relative response.

### Supplementary Information

Supplementary Information.

## Data Availability

The data and analysis code are provided as a supplemental file.
