# Supplementary material for: Validation of a modified visual analogue scale to measure user-perceived comfort of a lower-limb exoskeleton
Source: Sci Rep. 2023 Nov 22;13:20484. doi: 10.1038/s41598-023-47430-z (PMC10665473; doi:10.1038/s41598-023-47430-z)
Supplement: Supplementary file 1 — Supplementary Information. [file 41598_2023_47430_MOESM1_ESM.pdf]

Supplemental Figures and Tables

Supplementary Table 1. The minimum and maximum answer, the answer range, the repeatability coefficient (*RC*), and the percentage of trials with a consistent relative response (Cons) for each subject and question. The *RC* is often around the same magnitude as the range of the responses to each question.

	Question 1					Question 2				
Sub	Min (mm)	Max (mm)	Range (mm)	RC (mm)	Cons (%)	Min (mm)	Max (mm)	Range (mm)	RC (mm)	Cons (%)
1	28	60	32	32	44	30	60	30	33	11
2	33	46	13	13	56	28	51	23	17	44
3	53	80	27	19	60	52	78	26	19	60
4	21	99	78	57	22	15	90	75	82	67
5	42	91	49	46	38	47	91	44	40	38
6	13	100	87	76	38	0	100	100	85	38
7	7	69	62	36	89	4	78	74	49	67
8	49	100	51	55	44	41	89	48	56	33
9	34	55	21	17	22	13	48	35	31	33
10	51	77	26	26	44	21	73	52	55	56
11	35	72	37	35	44	42	72	30	19	22
12	24	66	42	30	44	34	73	39	32	44
13	12	41	29	26	44	12	46	34	23	67
14	46	71	25	19	78	15	86	71	37	67

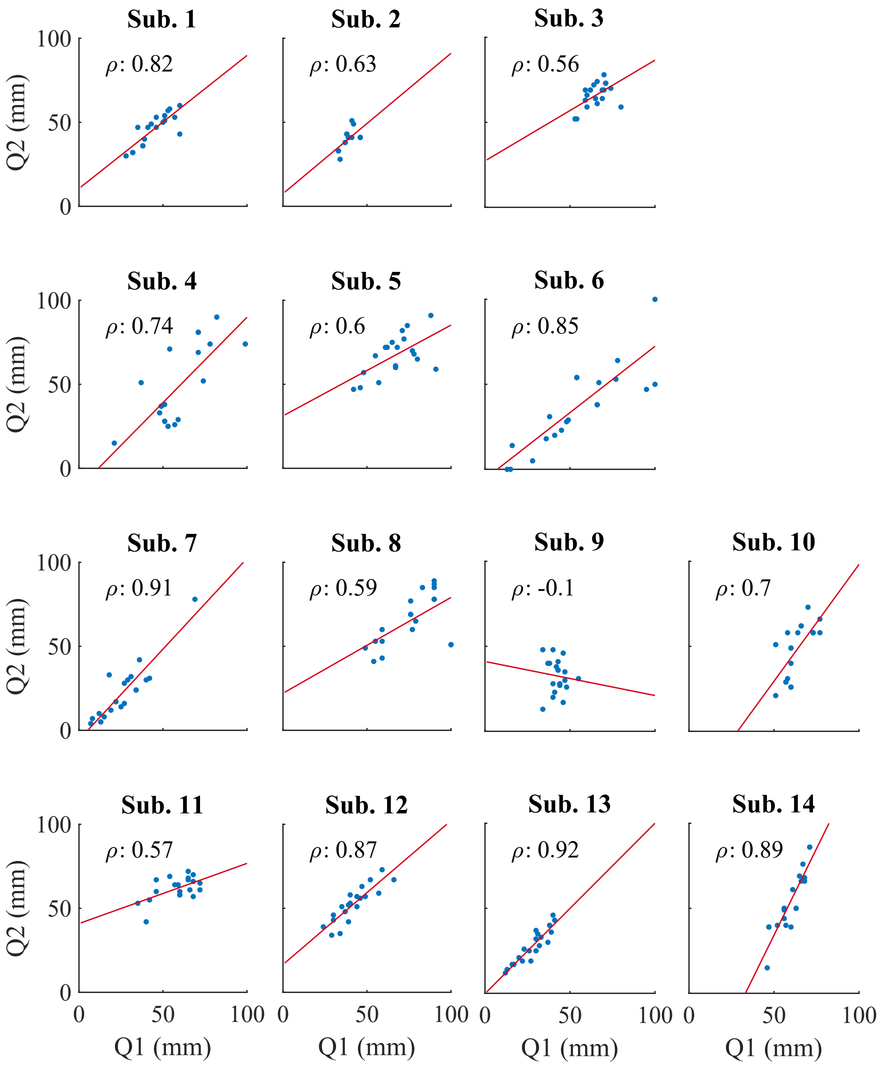

Supplementary Figure 1. Correlation plots between Q1 and Q2 for each subject. Most subjects displayed a strong positive correlation between both questions.

Supplementary Figure 2. Plots of normalized VAS answers (Eq. 4) for each subject and cycle. For 11 of the 14 subjects, the most comfortable control parameters changed between cycles 1 and 2. This implies that subjects were adapting to the exoskeleton over the course of the experiment, which in turn changed their perceived comfort.

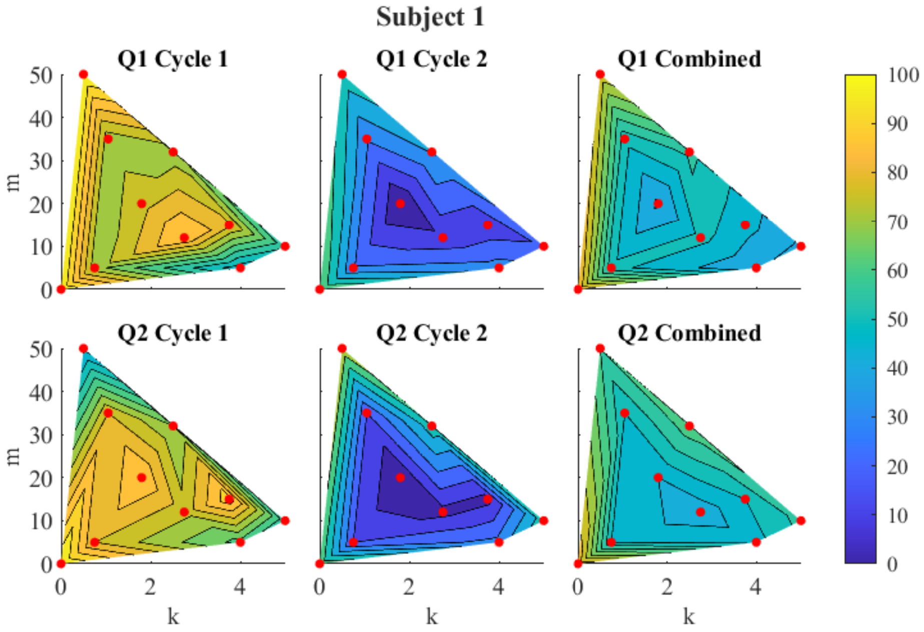

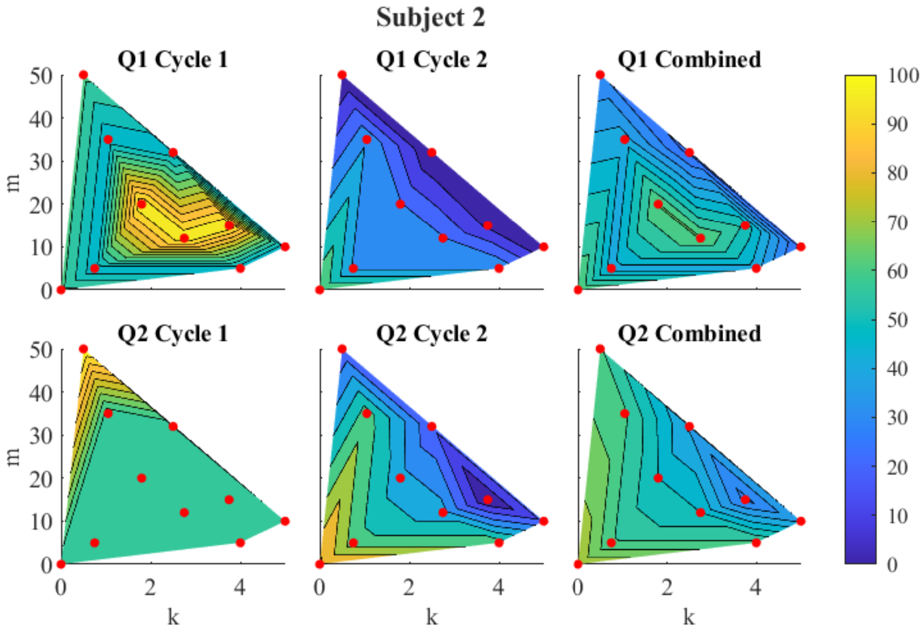

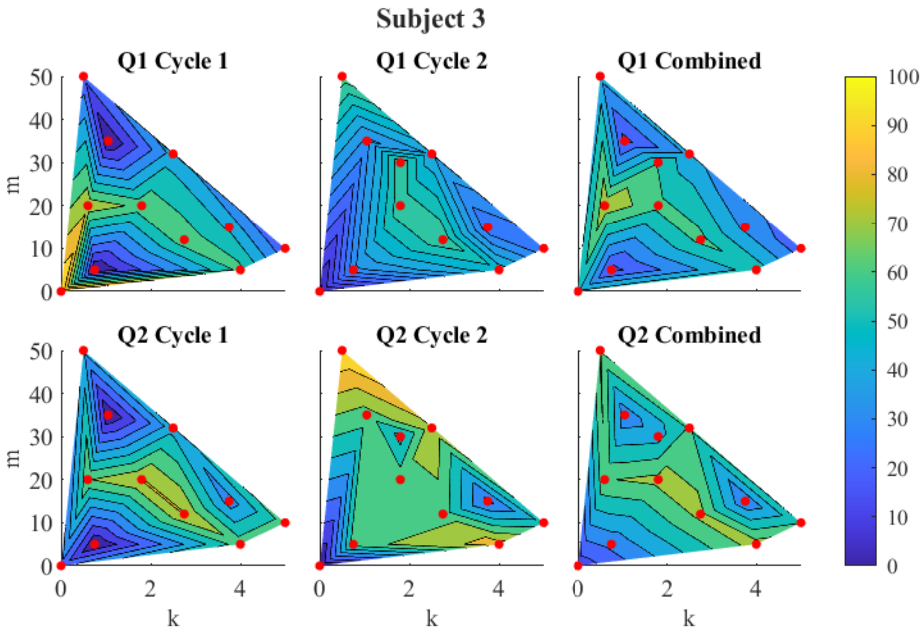
Subject 3
Q1 Cycle 1
Q1 Cycle 2
Q1 Combined
Q2 Cycle 1
Q2 Cycle 2
Q2 Combined
m
k
0
2
4
0
10
20
30
40
50
60
70
80
90
100

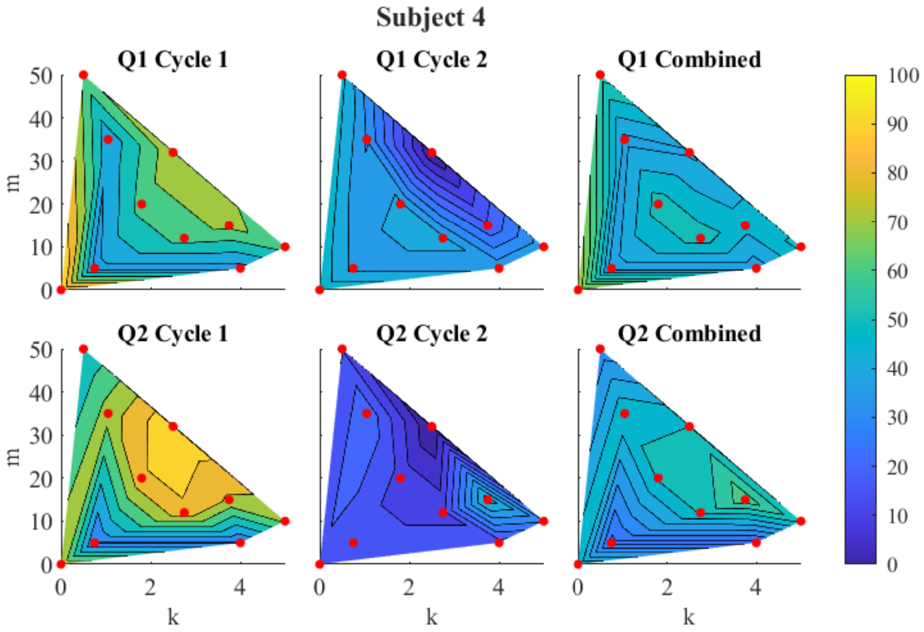
Subject 4
Q1 Cycle 1
Q1 Cycle 2
Q1 Combined
Q2 Cycle 1
Q2 Cycle 2
Q2 Combined
m
k
0
2
4
0
10
20
30
40
50
60
70
80
90
100

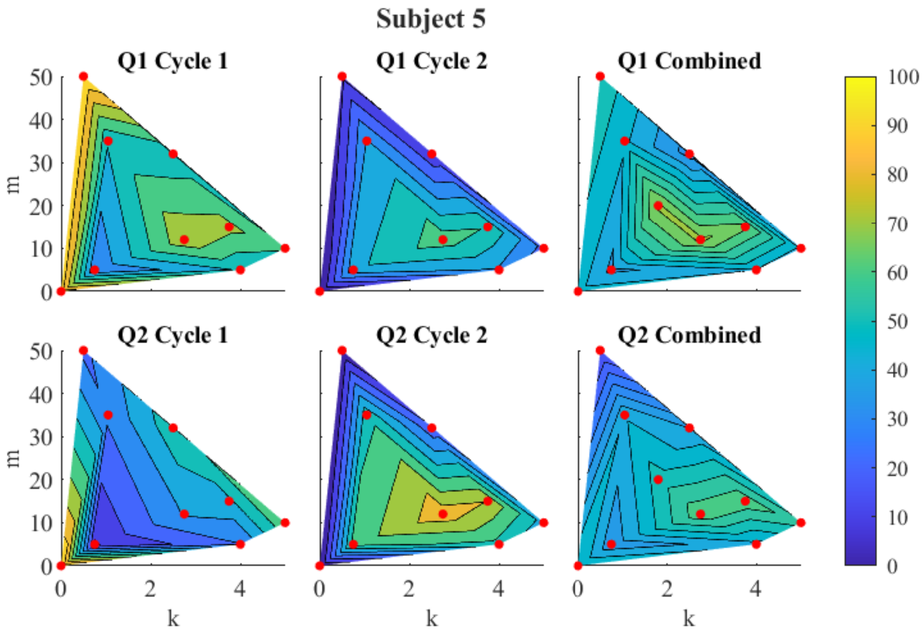
Subject 5
Q1 Cycle 1
Q1 Cycle 2
Q1 Combined
Q2 Cycle 1
Q2 Cycle 2
Q2 Combined
m
k
0
10
20
30
40
50
0
2
4
0
10
20
30
40
50
60
70
80
90
100

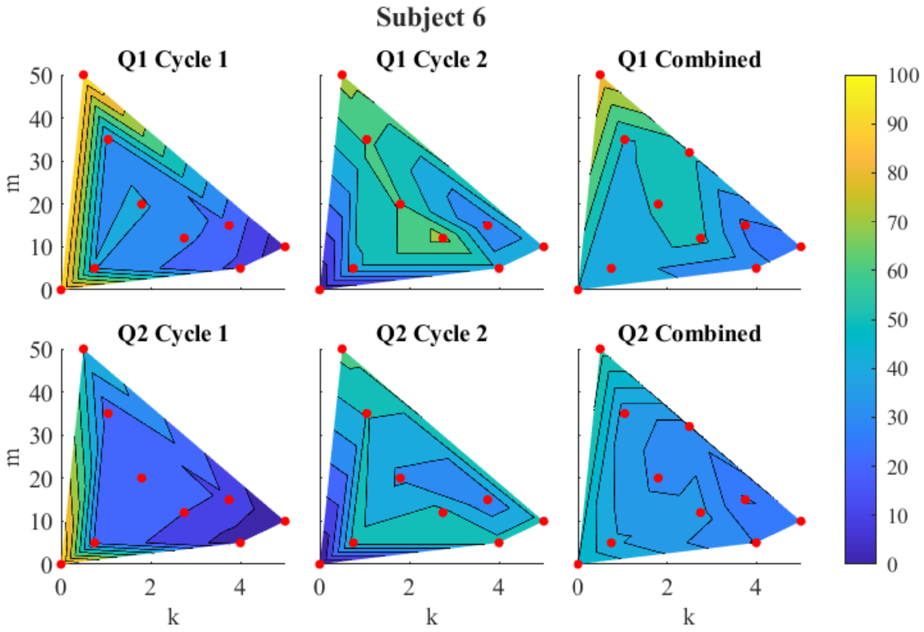
Subject 6
Q1 Cycle 1
Q1 Cycle 2
Q1 Combined
Q2 Cycle 1
Q2 Cycle 2
Q2 Combined
m
k
0
10
20
30
40
50
0
2
4
0
10
20
30
40
50
60
70
80
90
100

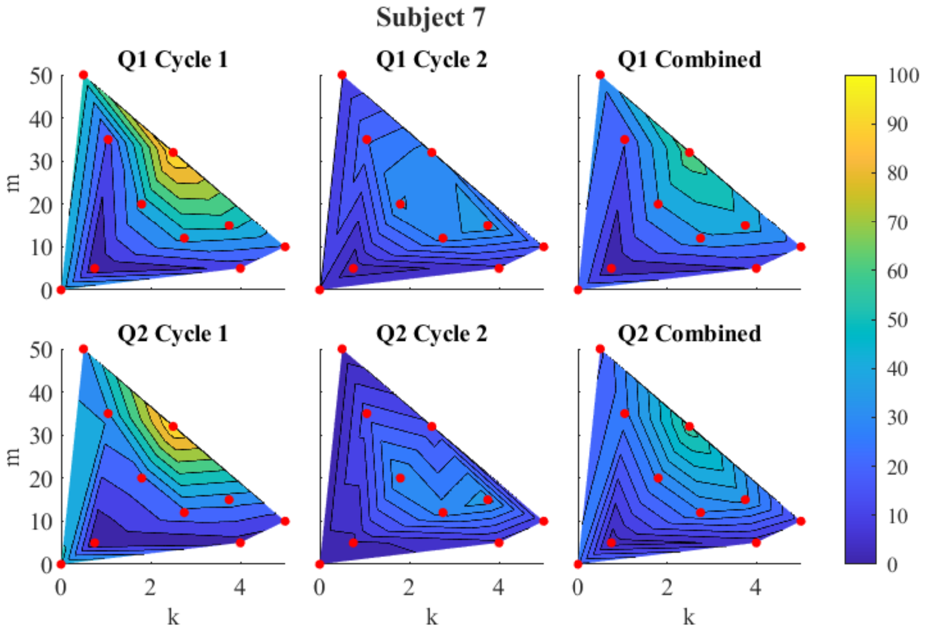
Subject 7
Q1 Cycle 1
Q1 Cycle 2
Q1 Combined
Q2 Cycle 1
Q2 Cycle 2
Q2 Combined
m
k
0
10
20
30
40
50
60
70
80
90
100

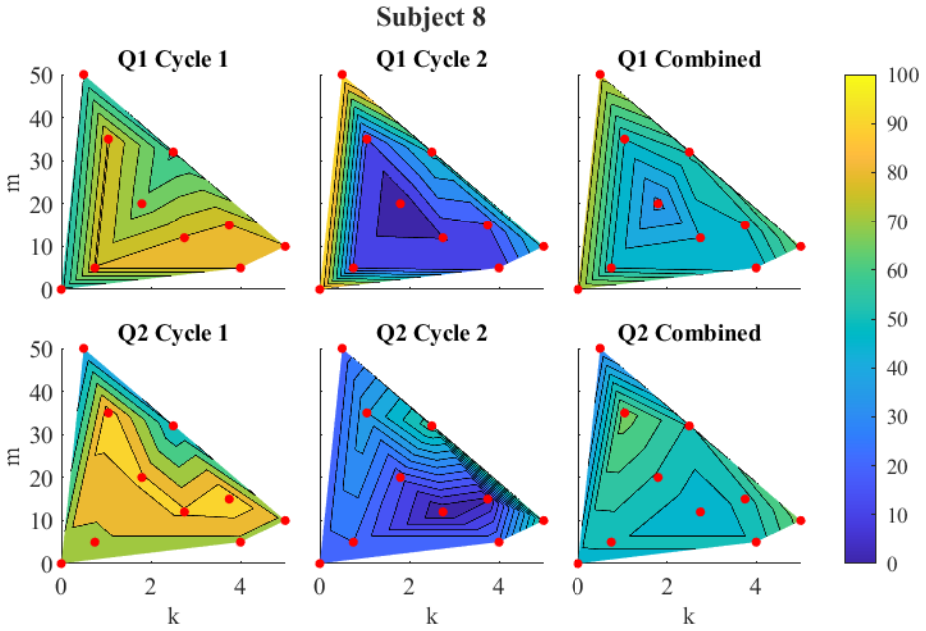
Subject 8
Q1 Cycle 1
Q1 Cycle 2
Q1 Combined
Q2 Cycle 1
Q2 Cycle 2
Q2 Combined
m
k
0
10
20
30
40
50
60
70
80
90
100

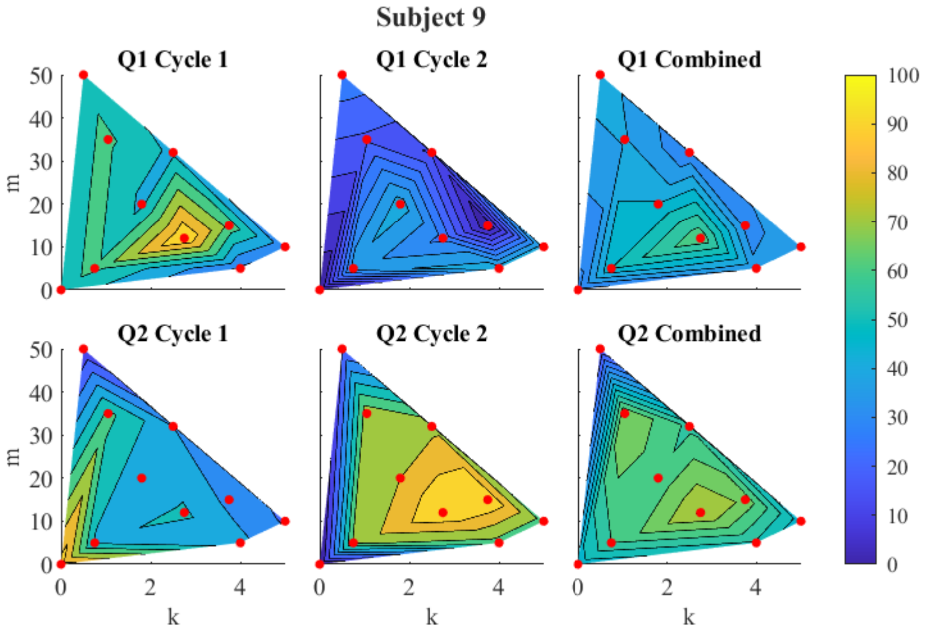
Subject 9
Q1 Cycle 1
Q1 Cycle 2
Q1 Combined
Q2 Cycle 1
Q2 Cycle 2
Q2 Combined
m
k
0 10 20 30 40 50
0 2 4
0 10 20 30 40 50 60 70 80 90 100

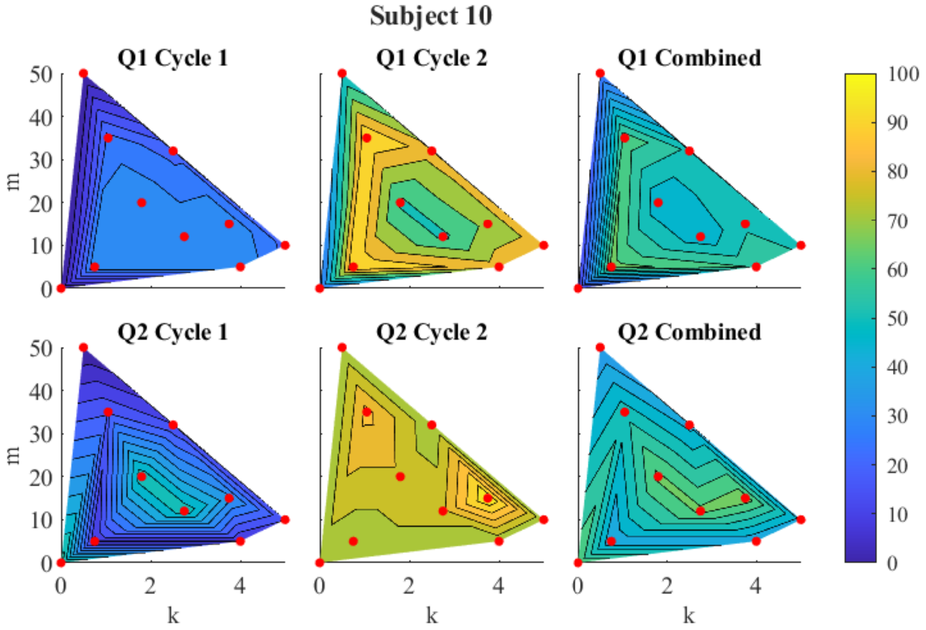
Subject 10
Q1 Cycle 1
Q1 Cycle 2
Q1 Combined
Q2 Cycle 1
Q2 Cycle 2
Q2 Combined
m
k
0 10 20 30 40 50
0 2 4
0 10 20 30 40 50 60 70 80 90 100

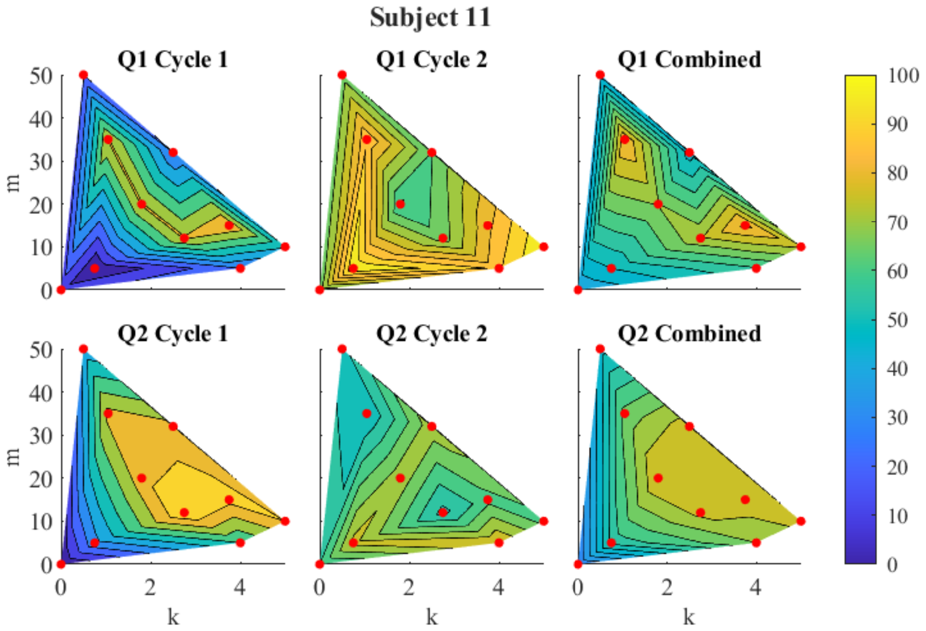
Subject 11
Q1 Cycle 1
Q1 Cycle 2
Q1 Combined
Q2 Cycle 1
Q2 Cycle 2
Q2 Combined
m
k
0
10
20
30
40
50
0
2
4
0
10
20
30
40
50
60
70
80
90
100

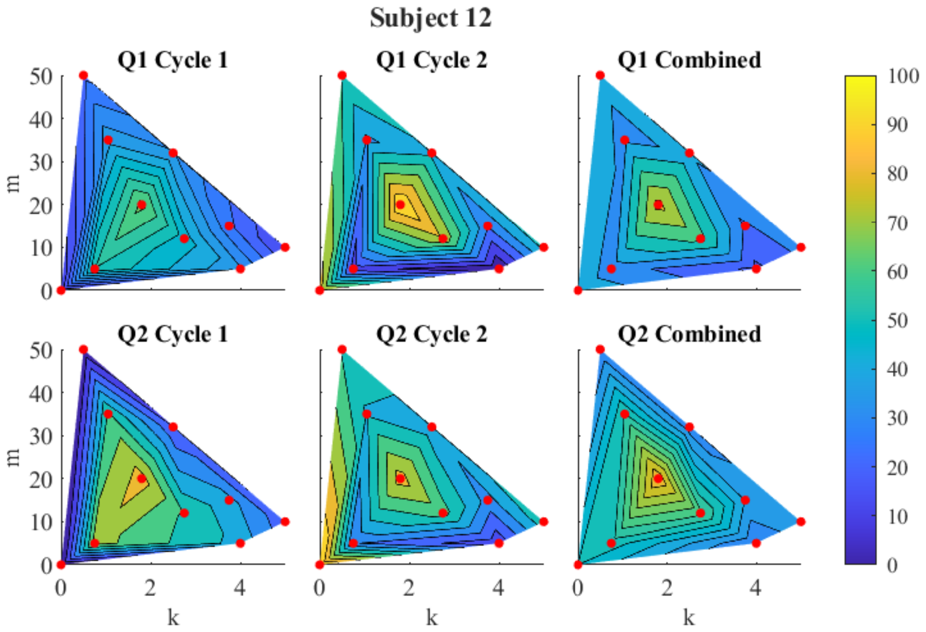
Subject 12
Q1 Cycle 1
Q1 Cycle 2
Q1 Combined
Q2 Cycle 1
Q2 Cycle 2
Q2 Combined
m
k
0
10
20
30
40
50
0
2
4
0
10
20
30
40
50
60
70
80
90
100

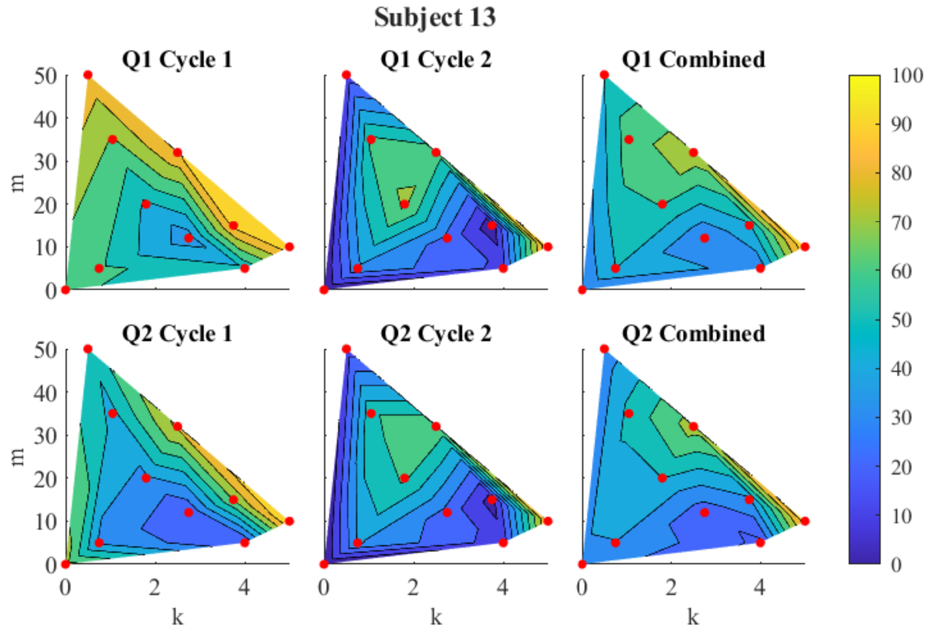

Subject 13
Q1 Cycle 1
Q1 Cycle 2
Q1 Combined
Q2 Cycle 1
Q2 Cycle 2
Q2 Combined
m
k

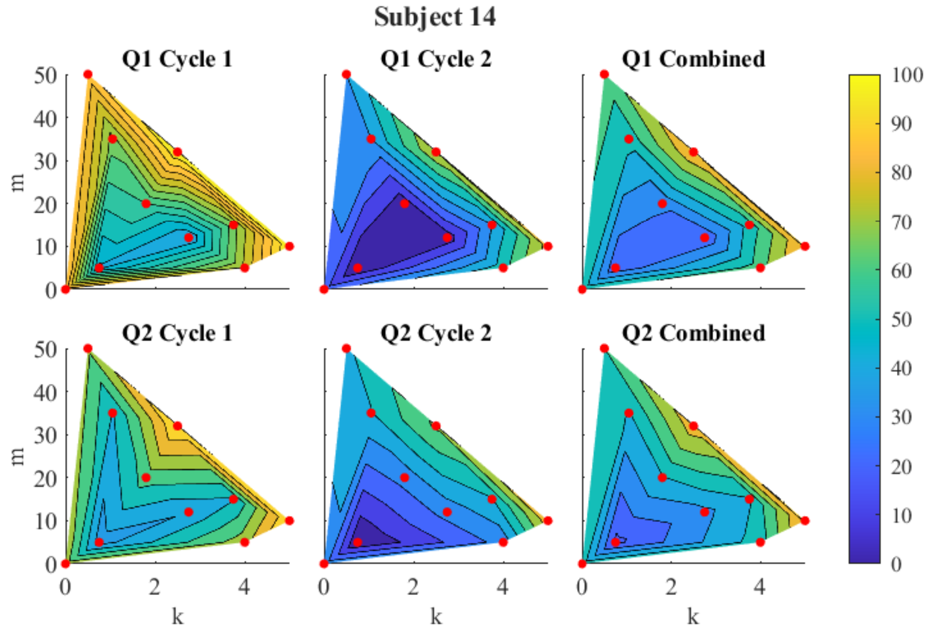

Subject 14
Q1 Cycle 1
Q1 Cycle 2
Q1 Combined
Q2 Cycle 1
Q2 Cycle 2
Q2 Combined
m
k

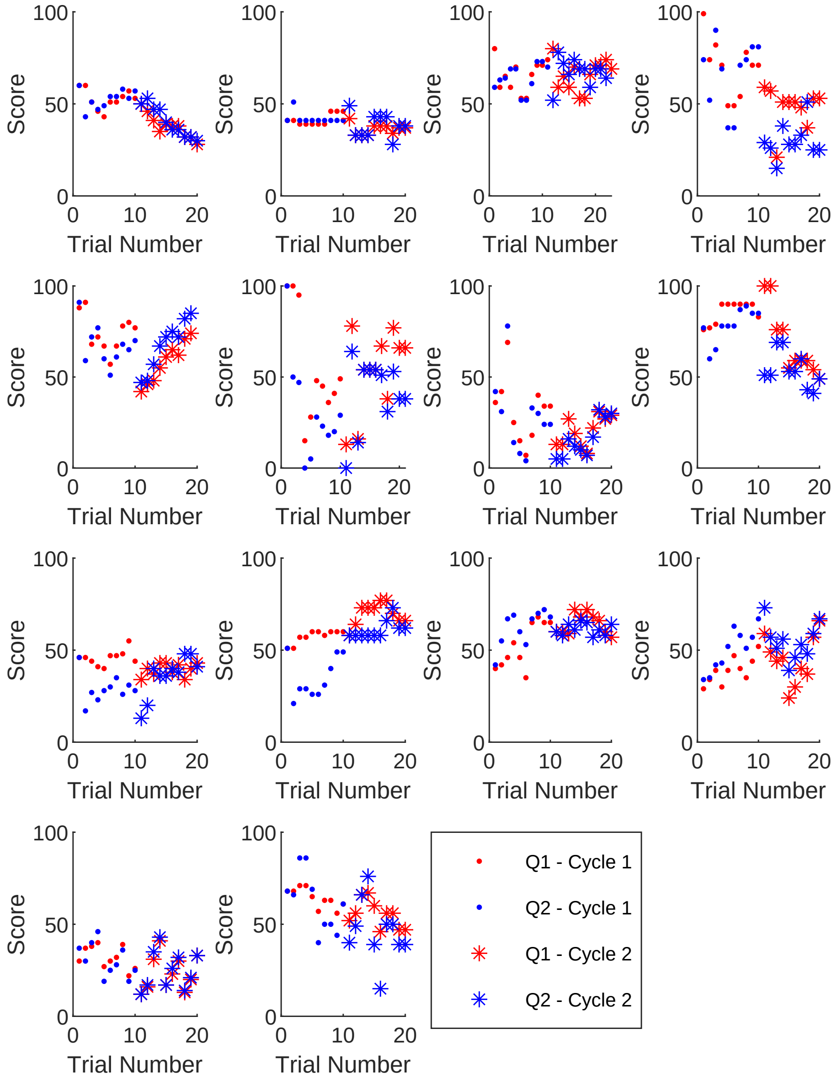

Supplementary Figure 3. Plots of question responses in sequential order. As expected, most subjects' answers did not consistently trend either up or down.
